# Upcycling of Mango Pulp and By-Products into Functional Flours: Effects on Bioactive Composition and Bioaccessibility in Cupcakes

**DOI:** 10.3390/foods15193527

**Published:** 2026-10-02

**Authors:** Francine Albernaz Lobo, Carla M. Stinco, Ana M. Benítez-González, Dolores Hernanz, María José Jara-Palacios

**Affiliations:** 1 Faculdade de Nutrição, Universidade Federal do Estado do Rio de Janeiro, Rio de Janeiro 22290-240, Brazil; francinealbernazlobo@gmail.com; 2 Food Color & Quality Laboratory, Facultad de Farmacia, Universidad de Sevilla, 41012 Sevilla, Spain; cstinco@us.es (C.M.S.); abenitez@us.es (A.M.B.-G.); 3 Department of Analytical Chemistry, Facultad de Farmacia, Universidad de Sevilla, 41012 Sevilla, Spain; mjara@us.es

**Keywords:** mango flours, bioactive compounds, phenolic and isoprenoids bioaccessibility, bakery systems, by-products valorization

## Abstract

The valorization of mango pulp and processing by-products as functional ingredients requires preserving bioactive compounds while ensuring their bioaccessibility after food processing and digestion. Five mango flours derived from pulp (freeze-drying, conventional drying and foam mat drying), peel and kernels were characterized and incorporated into cupcakes at a 35% wheat flour substitution level. The flours and cupcakes were evaluated for color, phenolic compounds and isoprenoids. Additionally, the bioaccessibility of these compounds was evaluated using the standardized INFOGEST in vitro digestion model. The pulp flours showed higher carotenoid content, whereas the kernel and peel flours were richer in galloyl derivatives and flavonols. After digestion, phenolic acids and mangiferin exhibited the highest bioaccessibility, while carotenoid and tocopherol bioaccessibility was strongly influenced by the drying process, with foam mat drying showing the highest values for lipophilic compounds. Overall, the results demonstrate that the nutritional functionality of mango-derived flours depends not only on their initial bioactive composition but also on the combined effects of mango part, drying process and food matrix on compound bioaccessibility. These findings support the use of mango-derived flours as sustainable functional ingredients for bakery applications.

## 1. Introduction

Mango (*Mangifera indica* L.) is one of the most harvested tropical fruits worldwide, and its industrial processing inevitably generates substantial amounts of by-products rich in valuable compounds [1]. These residues represent both a challenge and an opportunity, as they can be valorized to produce new high-value ingredients for the food industry, contributing to a circular economy and more sustainable manufacturing practices. The growing interest of the food sector in developing functional foods and implementing clean-label strategies positions mango by-products as promising raw materials for such applications [2].

Mango pulp is extensively used in industrial processing, leading to significant quantities of by-products, primarily peels and seeds and, to a lesser extent, leaves. These by-products may represent 35–60% of the total fruit weight, comprising approximately 15–20% peel and 35–40% seed [3]. Although mango kernels have received greater scientific attention due to their proportion and distinctive composition, both the kernel and peel have been identified as natural sources of bioactive compounds, often at higher concentrations than those found in the edible pulp [4].

Previous research shows that mango by-products can be successfully incorporated into bakery formulations, improving the fiber and bioactive content while maintaining acceptable sensory qualities [5,6,7]. Due to increasing environmental concerns, emerging processes aim to recover plant-based by-products and residues. Implementing innovative, cost-effective, and sustainable methods adds value to these materials, promoting their use in nutrition and healthy diets [8]. Mango kernels and peels possess antioxidant and antimicrobial properties, making them potential natural additives to extend food shelf life [9].

Mangiferin, a xanthone widely distributed in mangos, is of particular relevance due to its promising health benefits and potential applications in functional foods [10]. In this sense, the value of mango by-products should be highlighted, as they are related to a great challenge in the area of science and technology, with a potentially great scientific, technological and innovation impact [2].

These valorization efforts gain further relevance in the context of the United Nations’ 2030 Agenda, particularly Sustainable Development Goal (SDG) 12: Responsible Consumption and Production. Approximately one third of global food production becomes waste each year, generating severe economic and environmental impacts [11]. The fruit and vegetable processing industry is the third largest waste generator, followed by the dairy and beverage industries. An objective of the food industry in recent years has been to manage by-products and to encourage their appreciation as new raw materials [12]. In this way, the aim of this strategy is to reduce the negative ecological impact while making production as profitable as possible, thereby promoting an essential principle of sustainable economics [9].

The objective of this study was to investigate how the mango part (pulp, peel and kernels) and pulp drying method influence the composition and bioaccessibility of bioactive compounds after their incorporation into a bakery product. The phenolic compounds, isoprenoids and color were characterized in mango-derived flours obtained from different mango parts and drying processes. These flours were subsequently incorporated into cupcakes to assess the bioaccessibility of phenolic compounds and isoprenoids following standardized in vitro gastrointestinal digestion (INFOGEST), while consumer sensory acceptance was also evaluated.

## 2. Materials and Methods

### 2.1. Reagents

All solvents were HPLC-grade. Chromatographic solvent acetonitrile was purchased from Fluka (Madrid, Spain). Methanol (MeOH), methyl tert-butyl ether (MTBE), and ethyl acetate were supplied by Merck (Merck, Darmstadt, Germany) and Panreac (Barcelona, Spain). Ultrapure water (NANOpure Dlamond™ system, Barnsted Inc., Dubuque, IO, USA) was used for chromatography analyses. Enzymes and all other reagents used to prepare simulated oral fluid (SSF), simulated gastric fluid (SGF), and simulated intestinal fluid (SIF) were purchased from Sigma-Aldrich (St. Louis, MO, USA). Standards for carotenoids, chlorophylls, tocopherols and phenolic compounds were from Sigma-Aldrich (purity > 95%).

### 2.2. Samples

#### 2.2.1. Mango Samples

The mangoes used in this study, ripe Tommy Atkins fruits (*Mangifera indica* L. cv. Tommy Atkins), were purchased from a local supermarket in Brazil. Ripeness was determined based on the external peel color and manual firmness at the time of selection; the fruits showed a characteristic green–purple peel and firm texture typical of commercially ripe mangoes. The fruits were manually washed and peeled to obtain the pulp and by-products (kernel and peel). The pulp was processed using a blender (Metvisa^®^, 2.8 L 1200 W Inox Blender BLA, Brusque, SC, Brazil) and refined by sieving. The pulp samples were dried using three methods: (1) freeze-dried using an L108 lyophilizer (Liotop^®^, Liobras, São Carlos, SP, Brazil) at −54 °C; (2) dried in a conventional oven with forced-air ventilation at 80 °C for 10 h; and (3) dried with the foam mat drying technique using a concentration of 5% of albumin as the foaming agent, at a drying temperature of 80 °C for 4 h, and dryer air velocity of 1 m/s. Mango pulp was transformed into foam using a mixer (G. Paniz^®^, Caxias do Sul, Brazil). The foam was spread on trays, placed in an oven with forced ventilation, and dried to produce a porous film [13]. The drying process was concluded once the final water activity reached approximately 0.40, as measured using a digital water activity meter (Etec^®^, Digital Water Tester EC Meter, São Paulo, SP, Brazil).

The kernel and peel by-products were dried at 60 °C for 24 h and subsequently ground into a fine powder for further analysis. The dehydrated material was milled using a housemade blender (Vita450 Blender, Impomac^®^, Itajaí, SC, Brazil).

The resulting mango flours were LF (lyophilized flour), CF (conventional flour), FF (foam mat-dried flour), KF (kernel flour) and PF (peel flour); packaged in polyethylene bags; and stored in a desiccator for further analysis. The moisture content of the flours was 5.87 ± 0.84, 7.58 ± 0.51, 13.95 ± 1.14, 9.03 ± 0.23%, respectively.

#### 2.2.2. Cupcakes Samples

Based on a previously established standard formulation consisting of wheat flour with yeast (125 g), sugar (125 g), oil (130 g), eggs (2 units), and milk (30 mL), five cupcake formulations were developed by incorporating mango flours. In these formulations, wheat flour with yeast was partially replaced with the mango flours, while the remaining ingredients were kept unchanged. In addition, a control cupcake was prepared following the same standard formulation without the addition of mango flour.

Preliminary assays were conducted at substitution levels ranging from 10% to 50%, according to Thenabadu & Seneviratne [14]. A substitution level of 35% wheat flour was selected based on a preliminary sensory evaluation, which indicated better overall sensory acceptance. Once the batter was prepared, the cupcakes were baked in a 700 W waffle maker (Bestron Gofrer, ABWM300P, ’s-Hertogenbosch, The Netherlands) for 15 min.

The resulting cupcakes were coded according to the flour used: L-CK (lyophilized flour cupcake), C-CK (conventional flour cupcake), F-CK (foam mat-dried flour cupcake), K-CK (kernel flour cupcake), P-CK (peel flour cupcake) and CO-CK (control cupcake).

### 2.3. Color Measurement

The color of the mango flours and the cupcake samples was evaluated using a DigiEye imaging system [15]. The system includes a calibrated 10.2-megapixel Nikon D80 digital camera (Nikon Corporation, Tokyo, Japan) equipped with a Nikkor 35 mm f/2D lens (Nikon Corporation), a color sensor for display calibration, and an illumination box designed by VeriVide Ltd. (Leicester, UK).

For the objective color measurements, the mango flour samples were placed in 1 cm thick transparent plastic bottles. For the cupcakes, the external color was measured directly, whereas the internal color was assessed after cutting the cupcakes in half. The instrument was calibrated with standard black and white plates prior to analysis. Digital images were then captured to record the overall appearance of cupcakes at depths perceptible to consumers. During these measurements, the samples were illuminated using a diffuse D65 simulator. The CIELAB coordinates were obtained using DigiPix software (v.2.62 VeriVide Ltd., Leicester, UK).

In the CIELAB uniform color space, the psychophysical parameters chroma (C*_ab_) and hue (h_ab_) are defined as in the following Equation (1):

(1) $${C*}_{\mathrm{ab}}\;=\;\sqrt{{\left(a^{*}\right)}^{2}+{\left(b^{*}\right)}^{2}};\;h_{\mathrm{ab}}=\arctan\;\left(\frac{b^{*}}{a^{*}}\right)$$

Chroma (C*_ab_) is used to determine the degree of difference in a hue compared to a grey color with the same lightness, and is considered the quantitative attribute of colorfulness. Hue (h_ab_) is the attribute according to which colors are usually defined as reddish, greenish, etc.

### 2.4. Extraction of Bioactive Compounds in Flours and Cupcakes

For the extraction of phenolic compounds, 0.5 g of mango flours and cupcake samples were weighed, and 5 mL of 80% ethanol was added, according to the methodology described by Jara-Palacios et al. [16], with slight modifications. The mixture was homogenized by ultrasound at amplitude of 30% for 2 min and 20 kHz frequency, using a Q500 sonicator with a 1.6 mm probe (Qsonica, Newtown, CT, USA). The samples were immersed in an ice bath during sonication to cool the probe and prevent degradation due to heating, and then centrifuged (4190× *g* for 5 min). The supernatant was collected, and the residue was subjected to the same process twice. The liquid supernatants were combined and stored at −20 °C until UHPLC analysis. The extraction was carried out in triplicate.

For extraction of isoprenoids, the methodology proposed by Stinco et al. [17] was used. For this purpose, 0.15 g of mango flour samples and 1 g of cupcake samples were vigorously homogenized with 5 mL of dichloromethane: acetone: methanol (2:1:1) using a T25 Ultra-Turrax (IKA Works, Wilmington, NC, USA) at 24,000 rpm for 5 min. The mixture was centrifuged at 3900× *g* at 4 °C for 5 min. After that, the colored fraction was recovered, and the residue was submitted to the same extraction process until the color was exhausted. The colored fractions were combined and concentrated to dryness in a rotatory evaporator (Eppendorf Concentrator Plus, Hamburg, Germany). The extracts were dissolved in dichloromethane and saponified with 500 mL of KOH (30% *w*/*v* in MeOH) for 60 min in darkness and with gentle shaking. Any trace of the base was removed by NaCl 5% and water washing. The colored solutions obtained were concentrated to dryness as previously indicated. The extracts were redissolved in ethyl acetate and transferred into glass vials for RRLC analysis. The extraction was carried out in triplicate [17].

### 2.5. Simulated In Vitro Digestion

An in vitro static gastrointestinal model that simulates digestion was used for the cupcake samples, following the INFOGEST 2.0 protocol by Brodkorb et al. [18]. All parameters, such as electrolytes (composition of simulated fluids), enzymes, bile, dilution, incubation time, pH and temperature (similar to physiological conditions) and N_2_ (to avoid oxidation), were the same as those described in this protocol.

Once the digestion process was completed, the methodology described by Rodrigues et al. [19] was used to isolate the micelle-containing fraction. The resulting supernatants were centrifuged at 3900× *g* for 20 min at 4 °C using an Allegra X-12R centrifuge (Beckman Coulter, Inc., Brea, CA, USA), filtered through a 0.22 μm nylon membrane (Agilent Technologies, Santa Clara, CA, USA), and stored at −20 °C under a nitrogen atmosphere until analysis. All assays were performed in quadruplicate.

For the subsequent determination of carotenoids, 10 mL of micellar fraction was homogenized with 5 mL of diethyl ether and 0.5 g of NaCl using a T25 Ultra-Turrax at 24,000 rpm for 5 min. The mixture was centrifuged at 3900× *g* for 5 min at 4 °C, and the supernatant was collected. The residue was re-extracted under the same conditions until no color remained. All supernatants were pooled and concentrated to dryness in a rotary evaporator at a temperature below 30 °C. The resulting dried extracts were stored at −20 °C under a nitrogen atmosphere until analysis. Before the injection, the dry extracts were redissolved in ethyl acetate, centrifuged at 18,000× *g* for 5 min at 4 °C, and transferred to amber glass vials.

### 2.6. Calculation of Bioaccessibility

The bioaccessibility (%) was calculated as follows: (A/B) × 100, where A is the total content of bioactive compound in the supernatant (phenolic compounds) or in the micellar fraction (isoprenoids) after in vitro digestion, and B is the total content of bioactive compounds in the cupcake before digestion, expressed in the same units.

### 2.7. Analysis of Bioactive Compounds by Liquid Chromatography

The phenolic compounds were determined using an Agilent 1290 chromatograph (Agilent Technologies, Palo Alto, CA, USA) coupled to a DAD. The separation was performed according to Jara-Palacios et al. [20], using a C18 Eclipse Plus 120 column (1.8 μm, 50 × 2.1 mm) and a scan range set from 200 to 770 nm. The phenolic compounds were identified by their retention time and UV-vis spectroscopic characteristics using those of standards, with reference to the compounds previously identified in [21]. Quantification was carried out by external calibration of the areas of the chromatographic peaks obtained by DAD detection at 280 nm. The samples were analyzed in duplicate, and the results were expressed as mg polyphenol/100 g of dry matter. The total phenolic compounds were calculated as the sum of individual phenolic compounds. The isoprenoids were determined by RRLC using an Agilent 1260 chromatograph (Agilent Technologies, Palo Alto, CA; USA) equipped with a diode array detector (DAD) and a C30 YMC column (3 μm, 250 × 4.6 mm) (YMC, Wilmington, NC, USA) kept at 28 °C. The chromatographic conditions (linear gradient, flow rate, DAD conditions, etc.) were established following the validated method by Stinco et al. [17]. The carotenoids were identified based on the basis of their retention time and UV-vis spectra, and by comparison with our data library and standards when available. This approach was based on the method previously validated in [22], which enabled the identification of multiple carotenoid geometrical isomers and chlorophyll derivatives [23].

Quantification was achieved by external calibration, using curves obtained with standard carotenoids solutions. The results are expressed as mg isoprenoids/100 g of dry matter. The total isoprenoids content was calculated as the sum of the individual isoprenoids.

### 2.8. Sensory Analysis of Cupcakes

This study was carried out in accordance with the ethical guidelines of Comité de Ética de Investigación of the Universidad de Sevilla (CEIUS) (approval ID SICEIA-2024-00237). Prior to the sensory evaluation testing, all participants provided written informed consent for their participation in the sensory taste testing and the use of the data collected therefrom. The cupcakes made with mango flour were evaluated by eighteen department staff members with previous experience in sensory analysis.

The sensory evaluation was conducted in a suitable room at the Faculty of Pharmacy of the University of Seville, with adequate lighting and ventilation and isolated from external noise and odors. The cupcake samples were coded using randomly assigned three-digit numbers and presented in a randomized order. Before the evaluation, the panelists were informed about the nature of the products and the assessment procedure. The samples were evaluated for surface color, interior color, aroma, taste, and texture using a 10-point hedonic scale ranging from 1 (extremely dislike) to 10 (extremely like) [24]. Overall acceptability was assessed as the like/dislike response. Water was provided for palate cleansing between samples.

### 2.9. Statistical Analysis

A one-way analysis of variance (ANOVA) was applied to determine significant differences in the phenolic and isoprenoid levels of mango pulp flours, according to the drying method, and to evaluate differences in the phenolic and isoprenoid levels between undigested and digested samples in the bioaccessibility assays.

A one-way ANOVA was also used to evaluate the differences in the color parameters of dried mango pulp and by-product flours, as well as of the corresponding cupcake formulations, followed by Tukey’s post hoc multiple-comparison test.

An ANOVA was also applied to analyze the sensory attributes results. A statistically significant level was considered to be *p* < 0.05. In addition, correlations between the CIELAB color parameters and sensory evaluation scores were studied using simple regressions calculated by GLM. All statistical analyses were performed using StatSoft Statistica^®^ V 8.0 software.

## 3. Results and Discussion

### 3.1. Influence of Fruit Part and Drying Process on Bioactive Compounds in Mango Flours

The bioactive composition of mango flours was markedly influenced by both the mango part used (pulp, peel or kernel) and the drying process applied to the pulp. Distinct profiles of phenolic compounds and isoprenoids were observed among the flours, reflecting the combined influence of fruit part and processing conditions. The fruit part primarily determined the phenolic composition, whereas the drying method mainly affected the retention of carotenoids and other isoprenoids in the pulp flours. These compositional differences highlight the potential of mango-derived flours as functional ingredients with distinct nutritional properties.

#### 3.1.1. Phenolic Profile

The phenolic profile differed considerably among the mango parts. The kernel flour was characterized by the highest concentration of galloyl derivatives, whereas the peel flour contained larger amounts of flavonols and mangiferin. In contrast, the pulp flours showed a simpler phenolic profile dominated by phenolic acids. Similar distribution patterns were previously reported for mango tissues, reflecting the different physiological roles of these compounds within the fruit. A total of thirty compounds were identified and quantified, which were classified into four different phenolic groups: thirteen galloyl derivatives (galloyl glucose, gallic acid and four derivatives, galloylquinic acid, methyl gallate, methyl digallate ester, trigalloyl, tetragalloyl and pentagalloyl, and hexagalloyl glucose), nine phenolic acids (ellagic and *p*-hydroxybenzoic acids, coumaric acid glucoside, four coumaric acid glucoside isomers and two ferulic acid hexosides), seven flavonols (quercetin 3-*O*-glucoside, quercetin 3-*O*-rutinoside, quercetin galactoside, quercetin xyloside, quercetin, kaempferol and quercetin derivative) and one xanthone (mangiferin a C-glucoside-xanthone) (Table 1).

The phenolic profile of the mango flours revealed qualitative and quantitative differences. As shown in Table 1, galloyl derivatives were the predominant compounds in the KF and PF, and phenolic acids in the pulp flours (LF, FF, and CF).

In the KF, the most abundant galloyl derivate was methyl gallate (2848.28 mg/100 g), followed by methyl digallate ester (2341.91 mg/100 g) and galloyl glucose (1149.07 mg/100 g). High concentrations of gallic acid were also detected (768.25 mg/100 g). These results are consistent with previous studies that reported a high gallic acid content in mango kernels [9,25]. Other phenolic compounds detected in the KF included two phenolic acids, *p*-hydroxybenzoic acid and ellagic acid, and mangiferin. This type of flour was the only one that had ellagic acid (97.89 mg/100 g). This compound is particularly interesting due to its strong antioxidant activity [25].

Regarding the PF, four galloyl derivatives were detected, with galloyl glucose (255.67 mg/100 g) showing the highest concentration. The flavonol content in the PF was particularly noteworthy, with quercetin 3-*O*-glucoside (62.30 mg/100 g) and quercetin 3-*O*-rutinoside (49.08 mg/100 g) being the most abundant compounds, in agreement with the results reported by Alañón et al. [9]. Moreover, the PF showed the highest concentration of mangiferin (25.36 mg/100 g) among all mango flours. Luo et al. [26] reported that mangiferin is primarily located in the peel, with concentrations ranging from 0.04 to 7.34 mg/g in a Chinese mango variety. This phenolic xanthone glycoside is particularly interesting due to its wide range of bioactivities, including antioxidant, anti-inflammatory, antimicrobial, and antiviral effects [27].

In the pulp flours, the total phenolic compound content ranged from 106.01 mg/100 g in the FF to 127.71 mg/100 g in the CF, with phenolic acids being the predominant compounds. The main phenolic acid was ferulic acid hexoside, followed by *p*-hydroxibenzoic acid in the LF and FF, and coumaric acid glucoside isomer in the CF. Other phenolic compounds, such as methyl gallate, methyl digallate ester, and mangiferin, were detected but in lower concentrations (Table 1).

Considering the drying method, statistically significant differences (*p* < 0.05) were observed among the pulp flours, particularly in the content of *p*-hydroxybenzoic acid, coumaric acid glucoside isomer, and ferulic acid hexoside (Table 1). These differences are likely due to thermal processing, which may enhance the release of phenolic compounds bound to the food matrix [13,28].

#### 3.1.2. Isoprenoids

As observed for the phenolic compounds, both the fruit part and drying process influenced the isoprenoid profile. The pulp flours were the main source of carotenoids, whereas the peel and kernel flours contained considerably lower concentrations. However, the drying method markedly affected carotenoid retention, confirming the sensitivity of these compounds to processing conditions. A total of twenty-one isoprenoid compounds were identified in the mango flour samples, belonging to three different groups: two tocopherols (α and β-tocopherols), six pheophytins (pheophytin A and four isomers; pheophytin B and an isomer) and thirteen carotenoids (phytoene; α-, β- and five ζ-carotene isomers; lutein; zeaxanthin; and 13Z-β, 9Z-α and 9 Z-β-carotene) (Table 2).

As observed for the phenolic profile, the isoprenoids fractions also showed qualitative and quantitative differences among the mango flour samples analyzed. As shown in Table 2, the KF showed the lowest total isoprenoids content (0.48 mg/100 g), with only three compounds identified: α-tocopherols (0.44 mg/100 g), β-carotene (0.03 mg/100 g), and 9 Z-β-carotene (0.01 mg/100 g). In contrast, Souto et al. [29] reported a higher carotenoid concentration in Ubá mango kernel (9.68 mg/100 g).

As for the PF, it showed a total isoprenoids content of 3.24 mg/100 g, with a notable presence of β-tocopherol, lutein, zeaxanthin and pheophytins, compounds not detected in the other mango flours. The most abundant compound was α-tocopherols (1.81 mg/100 g), followed by β-carotene (0.33 mg/100 g) and β-tocopherols (0.32 mg/100 g). The total pheophytin content in the PF was 0.46 mg/100 g, with pheophytin a isomers representing the major fraction (0.37 mg/100 g), compared to pheophytin b and its derivative (0.09 mg/100 g). The presence of pheophytins was restricted to the mango peel (exocarp), which is consistent with the tissue-specific localization of chlorophyll. In this regard, variations in pheophytin *a* content have been reported to decrease from 2.67 mg/100 g in green, unripe mango to 0.71 mg/100 g in ripe fruit [30].

With respect to the carotenoids compounds, the total content in the PF was 0.65 mg/100 g, with β-carotene as the major carotenoid, followed by lutein (0.07 mg/100 g) and 9Z-β-carotene (0.07 mg/100 g), in agreement with Ranganath et al. [31], who found β-carotene and violaxanthin isomers as the predominant carotenoid components in mango peels. According to the peel color, mango cultivars are commonly classified into red, yellow, and green types [32,33]. Ranganath et al. [31] reported carotenoid concentrations ranging from 0.155 to 3.118 mg/100 g in yellow peels and from 0.317 to 1.219 mg/100 g in green peels, both of which exhibited higher carotenoid contents than red peels (0.074–0.627 mg/100 g), such as those of the Tommy Atkins cultivar.

According to Table 2, the total isoprenoids contents in the mango pulp flours ranged from 2.81 mg/100 g in the FF to 4.62 mg/100 g in the CF. Pheophytins were not detected in any mango pulp flour, and α-tocopherol was the only tocopherols present. It was the most abundant compound in the LF and CF (1.48 and 1.76 mg/100 g, respectively). Significant differences (*p* < 0.05) were observed depending on the drying method. These differences were significant in the levels of α-tocopherol, ζ-carotene isomer 5, α-carotene, 13Z-β-and 9Z-β-carotene (Table 2).

The yellow coloration observed during mango ripening is associated with carotenoid accumulation in the mesocarp, which corresponds to the edible pulp [34]. On this basis, the total carotenoids content in the mango pulp flours ranged from 1.78 mg/100 g in the LF to 2.86 mg/100 g in the CF. Similar results were reported by Maldonado-Celis [35] for Brazilian mango pulp from different varieties (Haden, Tommy Atkins, Ubá and Palmer), where the total carotenoids content ranged from 1.91 mg/100 g in cv. Haden to 2.63 mg/100 g in cv. Palmer. In the CF, the carotenoid content was higher than in the other pulp flours, which may be attributed to the disruption of cell structures and carotenoid–protein complexes during the conventional drying process, thereby increasing carotenoid release and availability [31]. The main carotenoids were ζ-carotene isomer 3 and β-carotene, with values ranging from 0.82 mg/100 g (CF) to 0.68 mg/100 g (LF) for ζ-carotene isomer 3, and from 0.77 mg/100 g (CF) to 0.43 mg/100 g (FF) for β-carotene. These amounts are consistent with those reported by Fratianni et al. [36], who highlighted β-carotene as the main carotenoid contributing to the nutritional value of mango.

#### 3.1.3. Color of Mango Flour

The color parameters reflected the compositional differences among the mango flours and the impact of drying conditions. Conventional drying produced darker flours with higher chroma values, whereas the freeze-dried samples retained lighter colors. The kernel flour exhibited the lowest color saturation, consistent with its lower carotenoid content. These color differences were consistent with the isoprenoid profiles observed among the flours.

**Figure 1:**
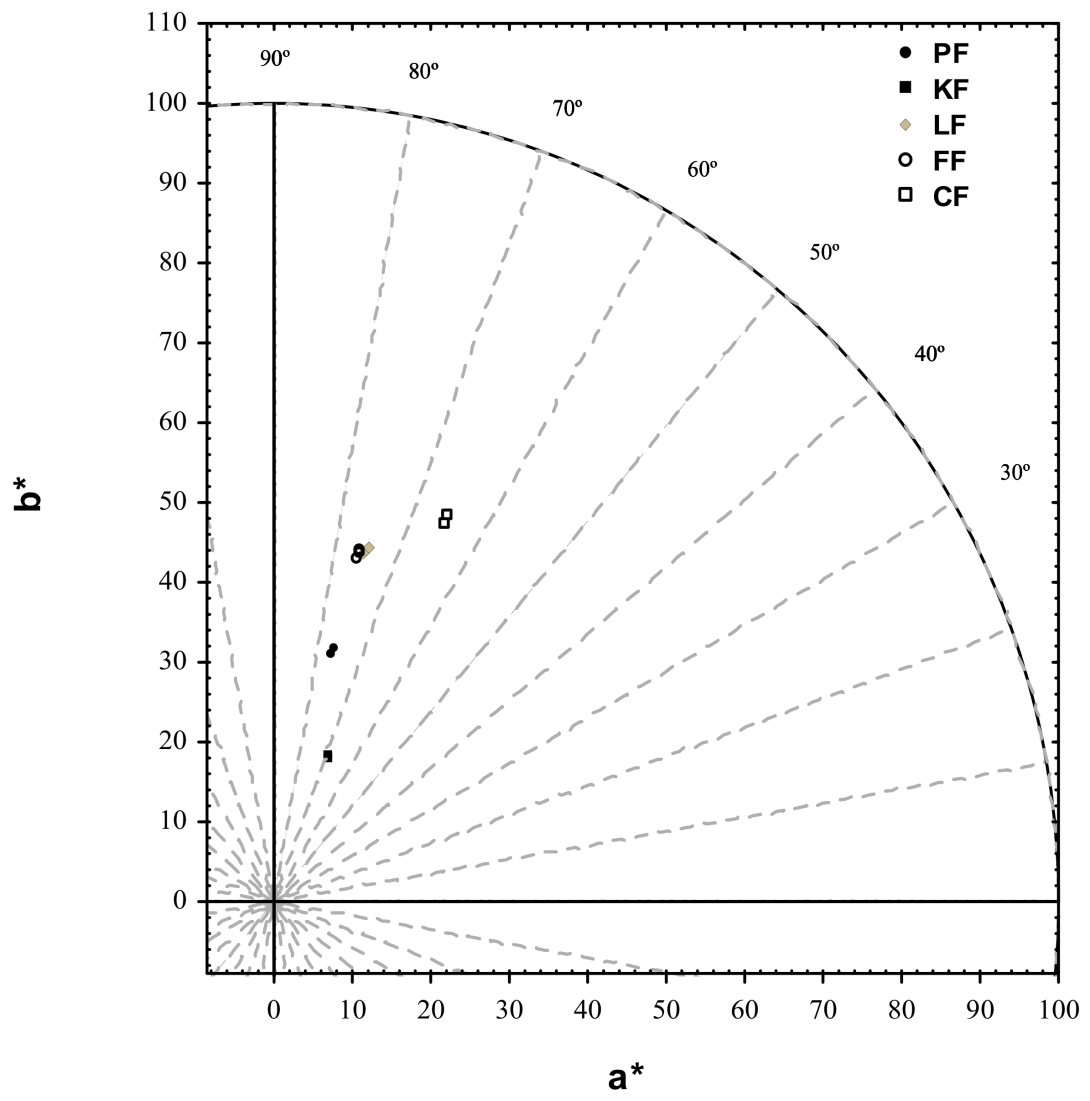
Localization of mango flours in the a*b* plane, as measured by digital image analyses using a DigiEye imaging system. Mango-derived flour: KF = kernel; PF = peel; LF = freeze-dried; FF = foam mat drying; CF = conventional dried.

Figure 1 shows the CIELAB color space (a*b* plane), showing the color of the mango flours analyzed in this study using digital image analyses. The L* coordinate values ranged from 51.77 for the CF (the darkest) to 78.27 for the LF samples (the lightest). The hue (h_ab_) allows for the differentiation of a color relative to a grey of the same lightness, is associated with variations in absorbance at different wavelengths, and is considered the qualitative attribute of color.

The results show little variation among the flours, with all samples falling within the first quadrant, between a 60° and 80° hue angle. Thus, the hue values ranged from 65° for the CF samples (the most reddish flours) to 77° in the PF samples (the most yellowish). Regarding chroma (C*_ab_), which represents color intensity and is measured as the distance from the origin (illuminate point) in the coordinates system, the values ranged from 19.38 in the KF samples to 52.46 in the CF samples.

Supplementary Table S1 presents the mean values of the CIELAB colorimetric parameters for the mango flours studied. Significant differences (*p* < 0.05) were found regarding the color when all mango flour were considered together. CF showed the highest a*, b*, and C*_ab_ values, and the lowest lightness and hue angle values. On the other hand, KF exhibited the lowest a*, b*, and C*_ab_ values.

### 3.2. Effect of Mango Part and Drying Process on Bioactive Compound Bioaccessibility

The bioaccessibility of bioactive compounds differed markedly among the cupcakes depending on both the mango flour incorporated and the drying process applied to the pulp flours. Overall, phenolic acids showed higher bioaccessibility than flavonoids and xanthones, whereas carotenoids and tocopherols exhibited a more variable behavior, strongly influenced by the flour type and processing conditions. These findings indicate that both the mango part and the technological treatment prior to baking determine the release of bioactive compounds during gastrointestinal digestion.

#### 3.2.1. Phenolic Compounds

**Figure 2:**
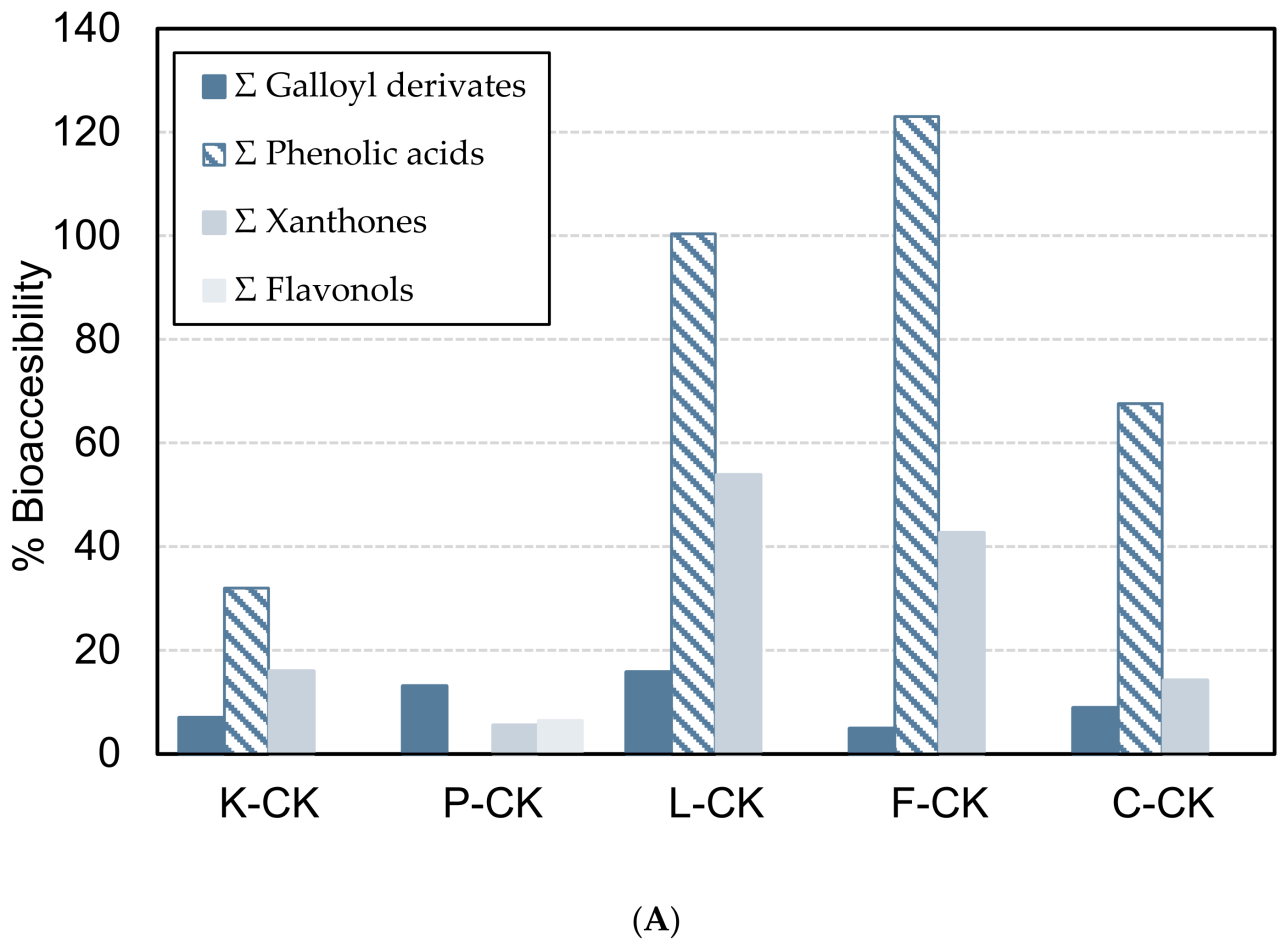

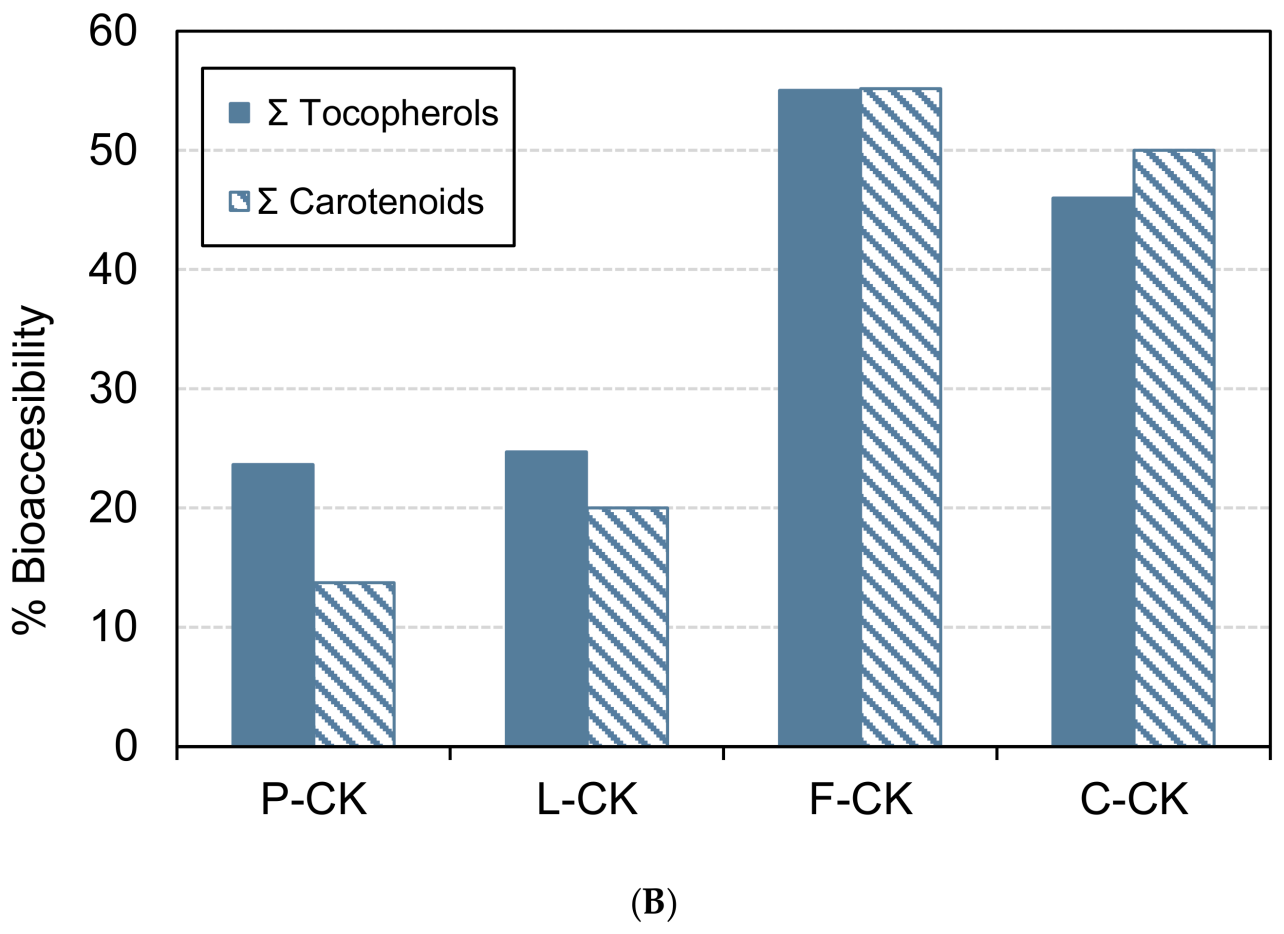
(**A**). Bioaccessibility percentage of phenolic compounds. K-CK = cupcake made with mango kernel; P-CK = cupcake made with mango peel; L-CK = cupcake made with lyophilized mango pulp; F-CK = cupcake made with foam mat-dried mango pulp; C-CK = cupcake made with conventional dried mango pulp. (**B**). Bioaccessibility percentage of tocopherols and carotenoids compounds. K-CK = cupcake made with mango kernel; P-CK = cupcake made with mango peel; L-CK = cupcake made with lyophilized mango pulp; F-CK = cupcake made with foam mat-dried mango pulp; C-CK = cupcake made with conventional dried mango pulp.

The phenolic compounds exhibited markedly different bioaccessibility depending on their chemical structure. Phenolic acids generally showed the highest recovery after digestion, whereas flavonols and gallotannins displayed considerably lower bioaccessibility. In this work, for the bioaccessibility, the phenolic profile of cupcakes before and after digestion was considered. Both the total contents of four phenolics groups (galloyl derivatives, phenolic acids, flavonols and mangiferin) and the content of individual phenolic compounds were considered. These results are shown in Table 3 and Table S2, and Figure 2A.

As expected, the cupcakes presented a phenolic profile consistent with the phenolic profile of the added flours. In K-CK and P-CK, galloyl derivates were the predominant group and phenolic acids were prominent in L-CK, F-CK and C-CK. Considering the phenolic contents of each phenolic group, there were significant differences (*p* < 0.05) before and after digestion for the five types of samples (Table 3). In general, the phenolic content decreased in the bioaccessible fraction with respect to the undigested sample, except for the phenolic acids in L-CK and F-CK, where there was a slight increase (*p* < 0.05), due to the increased content of *p*-hydroxybenzoic acid. This increase may be due to hydrolysis of polymerized compounds present in the pulp. Previous studies have also reported an increase in some phenolic acids after digestion, indicating that this could be due to the action of digestive enzymes that promote the liberation of phenols or hydrolyze protein–polyphenol complexes [37,38,39].

The bioaccessibility ranged from 4.86% to 123.00%, with phenolic acids showing the highest values (31.97–123.00%), followed by mangiferin (5.52–53.81%), galloyl derivatives (4.86–15.77%), and flavonols (6.45%) (Figure 2A).

In K-CK, the most bioaccessible phenolic group was the phenolic acids (31.97%), followed by mangiferin (15.95%) and galloyl derivates (6.96%). Although these last compounds were the most abundant in this type of cupcake, their bioaccessibility was low; the total content decreased from 3636.03 to 253.01 mg/100 g. Among them, the most bioaccessible compound was D1. gallic acid (41.20%) and the least bioaccessible were methyl gallate, methyl digallate ester and trigalloyl glucose (2.16, 2.51 and 2.69%, respectively). This can be explained by chemical reactions, such as hydrolysis and deglycosylation that occur during gastric digestion and within the small intestine. Generally, monomeric polyphenols are primarily absorbed in the small intestine. As a result, the portion of phenolic compounds that is not absorbed reaches the large intestine, where it is further metabolized by microbial enzymes [40]. A relevant compound in K-CK was gallic acid (601.51 mg/100 g); however, it showed low bioaccessibility (20.23%).

The two phenolic acids detected in K-CK, ellagic acid and *p*-hydroxybenzoic acid, were decreased in the digested samples (from 63.45 to 20.06 and from 7.32 to 2.56 mg/100 g, respectively), with bioaccessibility being 31.61 and 34.97%, respectively.

Considering P-CK, the galloyl derivates groups showed higher bioaccessibility than flavonols and mangiferin (13.06, 6.45 and 5.52%, respectively). Galloyl glucose and gallic acid were the most abundant compounds in these cupcakes and showed a bioaccessibility of 21.03 and 6.66%, respectively. Among the flavonols, the quercetin glucoside stands out, which had a concentration of 19.31 and 3.07 mg/100 g before and after digestion, respectively, assuming a bioaccessibility of 15.89%.

In the case of cupcakes made with pulp (L-CK, F-CK and C-CK), the total content of phenolic groups showed the same trend (Figure 2A): the group of phenolic acids was the most bioaccessible (between 67.59 and 123.00%), followed by xanthone mangiferin (between 14.17 and 53.81%) and galloylated derivatives (between 4.86 and 15.77%). The contents of individual phenolic compounds were affected by the digestion process, generally resulting in a decrease. However, *p*-hidroxybenzoic acid had a different trend to the rest of compounds in all the cupcakes made with pulp; the content of this compound was significantly higher in the micellar fraction than in the undigested sample, with 272.14, 359.84 and 180.88% bioaccessibility in L-CK, F-CK and C-CK, respectively. Comparing the total phenolic compounds in the three types of cupcakes made with pulp, no significant differences were found between L-CK, F-CK and C-CK either before or after digestion.

It is noteworthy that the phenolic acids and mangiferin exhibited higher bioaccessibility in the cupcakes made with dried mango pulp (67.59–123.00% and 14.17–53.81%, respectively) than with kernels (31.97% for phenolic acids and 15.95% for mangiferin) and peels (5.52% for mangiferin). This may be attributed to the interaction between the polyphenols and macronutrients within the food matrix, which influences the bioavailability of mango polyphenols. Previous studies have shown that fiber can influence the absorption of phenolic compounds in the small intestine [41,42]. Consequently, processing techniques, such as heat treatments, milling, fermentation, and homogenization, can enhance the bioaccessibility and bioavailability of bioactive compounds from complex food matrices [43].

The matrix effect is important for bioaccessibility but also for bioavailability. Quirós-Sauceda et al. [44] investigated the effect of matrix (juice vs. pulp) on the bioavailability of mango phenolic compounds in humans. The group consuming mango juice exhibited a higher concentration of some phenolic acids in their plasma and urine compared to the pulp groups. These findings suggested that the processing of mango pulp into juice can improve the bioavailability of phenolic compounds.

Although further studies are required to establish the in vivo bioactive effects of mango-based cupcakes, these food products could be considered as a rich source of phenolic compounds, and their processing could allow for the economic and sustainable revaluation of mango by-products.

#### 3.2.2. Carotenoids and Tocopherols

Table 4 and Table S3 show the bioaccessibility of tocopherols and carotenoids in the cupcakes formulated with dried mango pulp and mango peel flours. Pheophytins were not detected in the micellar fraction. Moreover, isoprenoids were not detected in the micellar fraction of cupcakes prepared with mango kernel flour (K-CK).

In all the cupcakes, tocopherols were the predominant bioactive group. Overall, the tocopherol contents decreased in the micellar fraction compared with the undigested samples. Significant differences (*p* < 0.05) in tocopherols were observed before and after digestion across the four cupcake formulations (Table 4). The tocopherol contents in the undigested samples and micellar fractions (mg/100 g) varied from 0.32 to 0.03 in P-CK, from 0.18 to 0.02 in L-CK, from 0.12 to 0.05 in F-CK and from 0.17 to 0.04 in C-CK for *β*-tocopherol, and from 6.87 to 1.67 in P-CK, from 4.88 to 1.23 in L-CK, from 4.79 to 2.63 in F-CK, and from 5.83 to 2.72 in C-CK for *α*-tocopherol. The bioaccessibility of *α*-tocopherol tended to be higher than that of *β*-tocopherol. The bioaccessibility ranged from 9.38% to 41.67% for *β*-tocopherol and from 24.31% to 55.37% for *α*-tocopherol, corresponding to P-CK and F-CK, respectively. These results are consistent with previously reported bioaccessibility ranges of 46–81% for *α*-tocopherol in durum wheat and egg pasta [45], and 33.5% in milk–fruit juice beverages [17]. Kim et al. [46] indicated that several intrinsic and extrinsic factors can modulate tocopherol bioaccessibility, including food matrices; the presence of other nutrients, such as fiber, fats, and phytosterols; and processing techniques.

Regarding the carotenoid content, a decrease was observed in the micellar fraction compared with the undigested samples. Significant differences (*p* < 0.05) were observed before and after digestion across the four cupcake formulations, except for ζ-carotene isomer 3 and *α*-carotene (Table 4). Thus, the carotenoid contents in the undigested samples and digesta (mg/100 g) ranged from 0.51 to 0.07 in P-CK, from 0.35 to 0.07 in L-CK, from 0.29 to 0.16 in F-CK, and from 0.38 to 0.19 in C-CK.

Figure 2B shows the bioaccessibility of tocopherols and carotenoids in the cupcakes prepared with mango pulp and peel flours. Carotenoids bioaccessibility ranged from 13.73% to 55.17%, corresponding to P-CK and F-CK, respectively, while tocopherols bioaccessibility ranged from 23.64% to 55.03%, also corresponding to P-CK and F-CK.

Overall, bioaccessibility was lower in the cupcakes prepared with mango peel flour than in those formulated with mango pulp flour. Heat treatments associated with drying processes are known to enhance bioaccessibility by promoting carotenoid release from the food matrix through disruption of cellular structure. In particular, the foam mat drying method showed the highest bioaccessibility of both tocopherols and carotenoids, which may be attributed to matrix disruption, increased surface area, and enhanced release of lipophilic compounds, as previously reported for foam mat-dried fruit matrices [28,47,48].

Collectively, these results demonstrate that bioaccessibility is governed not only by the concentration of bioactive compounds in the raw material but also by the interactions between flour composition, drying process and the bakery matrix. Therefore, selecting appropriate processing conditions is essential to maximize the nutritional functionality of mango-derived flours intended for food applications.

### 3.3. Sensory Acceptability of Cupcakes Enriched with Mango Flours

#### 3.3.1. Sensory Analysis

Consumer acceptance is a key requirement for the successful incorporation of functional ingredients into bakery products. Therefore, the sensory attributes of cupcakes formulated with mango-derived flours were evaluated to determine whether the incorporation of pulp and by-product flours affected product acceptability. The overall acceptance scores remained high for all formulations despite significant differences in some individual sensory attributes. The cupcakes containing pulp flours had sensory profiles comparable to the control, whereas the peel and kernel flours produced slight changes in color and flavor that did not substantially reduce consumer acceptance.

**Figure 3:**
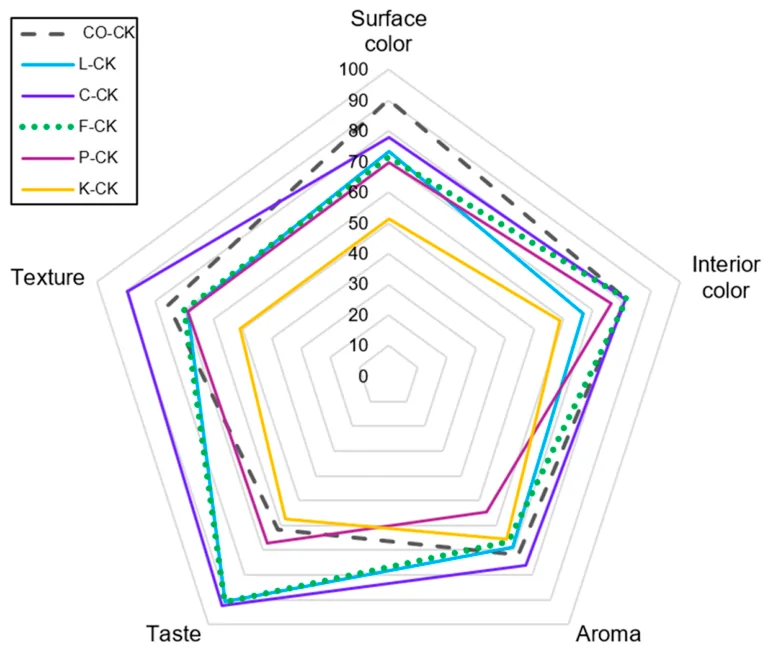
Mean scores of the sensory analysis for control (CO-CK) and fortified cupcakes (K-CK, P-CK, L-CK, F-CK, and C-CK).

Figure 3 presents the mean scores for texture, surface color, interior color, taste, and aroma of the control cupcakes and those made with dried mango pulp and by-products. Significant differences (*p* < 0.05) were observed between the supplemented cupcakes for the attributes of surface color, interior color, taste and texture. In contrast, no significant differences were found for the aroma attribute, indicating that the panelists did not perceive noticeable variations in aroma between the different formulations (Table S5).

C-CK obtained the highest scores for aroma, taste, and texture (7.7, 9.2 and 8.9, respectively). Regarding exterior color, the control cupcakes (CO-CK) and C-CK and L-CK achieved the highest scores (9.0, 7.8 and 7.1, respectively). For interior color, F-CK, C-CK, and CO-CK had the greatest sensory acceptance. The preference for the interior color of F-CK may be attributed to the incorporation of foam and stabilizing agents during formulation. This characteristic, inherent to the foam mat drying technique, likely influenced the final product structure and appearance, contributing to improved consumer acceptance [47].

Considering the cupcakes made with mango by-products, P-CK showed noteworthy scores for taste and texture, while K-CK stood out for aroma. Although K-CK recorded the lowest scores for taste (5.7), texture (5.1), surface color (5.1) and interior color (5.9), these results still indicate potential for product valorization.

In terms of overall acceptability, the cupcakes made with dried mango pulp (F-CK, L-CK, and C-CK) received the highest scores, followed by P-CK and CO-CK, while K-CK was the least preferred. Similar trends have been reported in the literature, where cookies fortified with 20% mango kernel flour achieved high sensory acceptability, whereas the incorporation of more than 15% papaya seed powder negatively affected consumer acceptance of chocolate cupcakes [49,50].

From a practical perspective, the sensory results demonstrate that the incorporation of mango-derived flours, particularly those obtained from pulp, can improve the nutritional value of bakery products without compromising consumer acceptance [51,52]. Therefore, these flours represent promising functional ingredients capable of combining sustainable by-product valorization with acceptable sensory quality.

#### 3.3.2. Color of Cupcakes

Table S4 shows the mean values of CIELAB colorimetric parameters obtained for the cupcakes studied by the DigiEye imaging system. Both the internal and external colors are included, with internal variables denoted by subindex (i) and the external variables by subindex (e).

**Figure 4:**
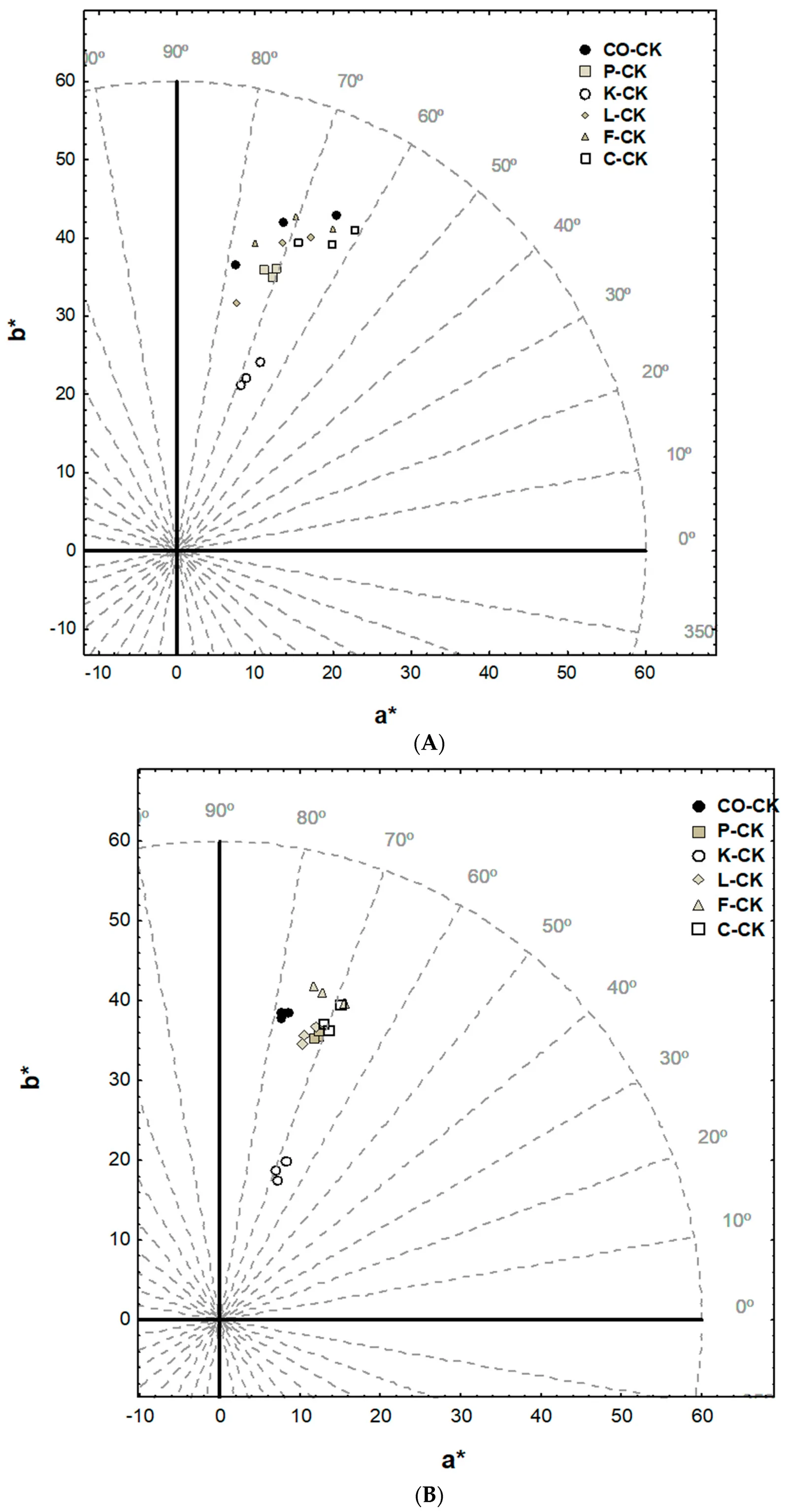
(**A**). Localization of *surface color* of cupcakes in the a*b* plane, as measured by digital image analyses using the DigiEye imaging system (control cupcake (CO-CK) and fortified cupcakes (K-CK, P-CK, L-CK, F-CK, and C-CK)). (**B**). Localization of *interior color* of cupcakes in the a*b* plane, as measured by digital image analyses using the DigiEye imaging system (control cupcake (CO-CK) and fortified cupcakes (K-CK, P-CK, L-CK, F-CK, and C-CK)).

Surface color: Significant differences (*p* < 0.05) were observed in the external color when considering all cupcake formulations. CO-CK exhibited the highest lightness and h_ab(e)_ values, while F-CK showed the highest b*_(e)_ and chroma values. In contrast, K-CK presented the lowest a*_(e)_, b*_(e)_ and chroma values. As shown graphically in Figure 4A, two distinct groups can be observed. One comprises the control cupcakes and those made with flours incorporating mango pulp and skin, and the other is formed by the cupcakes made with kernel flour, which are clustered closer to the origin of the coordinate plot, indicating the lowest color intensity.

Internal color: The internal color of the cupcakes also showed statistically significant differences (*p* < 0.05) when all formulations were considered. The distribution of the cupcakes grouped according to their formulation in the a*b* plane is plotted in Figure 4B. In this instance, the results followed a similar trend to those observed for the surface color. CO-CK exhibited the highest lightness and h_ab(e)_ values, while F-CK showed the highest b*_(e)_ and chroma values. In contrast, K-CK presented the lowest values for all colorimetric parameters, indicating that these cupcakes had the darkest color and the least vivid internal coloration.

Simple correlation analyses were performed between the CIELAB color parameters obtained from the DigiEye imaging system and the sensory evaluation scores. Significant correlations (*p* < 0.05) were found between the external L*_(e)_ and sensory surface color scores (*r* = 0.93), and between the internal b*_(i)_, C*_ab(i)_ and sensory interior color scores (*r* = 0.86 and 0.85, respectively).

## 4. Conclusions

This study highlights the potential of mango pulp and by-products, particularly kernels and peels, as valuable sources of bioactive compounds for the development of sustainable value-added foods. The mango by-product flours showed high phenolic contents, with the kernel flour rich in galloyl derivatives and the peel flour notable for flavonols and mangiferin, while the pulp-derived flours exhibited higher carotenoid levels, especially β-carotene. In the cupcakes, the phenolic acids were the most bioaccessible compounds, followed by mangiferin, whereas carotenoid and tocopherol bioaccessibility was strongly influenced by the drying method, with those made from the foam mat-dried pulp flour showing the highest values. The sensory evaluation demonstrated that cupcakes formulated with up to 35% mango pulp or by-product flours were well accepted, supporting the feasibility of these flours as biofunctional ingredients for sustainable food applications and waste reduction.

Although this study demonstrates the potential of foam mat drying for producing high-quality mango flours, some limitations should be acknowledged. Firstly, this research evaluated a single mango variety and a specific substitution level; hence, future studies should investigate different cultivars and a broader range of incorporation levels to optimize the formulation. Secondly, regarding industrial scalability, transitioning foam mat drying from laboratory to large-scale production requires optimizing foam stability, continuous-feed parameters, and energy efficiency.

## Figures and Tables

**Table 1:** Phenolic contents of flours obtained from mango dried pulp and by-products, expressed as mg/100 g on dry basis (mean ± SD; n = 3 *).

Mango Flour **	KF	PF	LF	FF	CF
Galloyl derivatives					
Galloyl glucose	1149.07 ± 39.84	255.67 ± 14.49	nd	nd	nd
Gallic acid	768.25 ± 35.65	85.64 ± 4.49	nd	nd	nd
D1. gallic acid	29.75 ± 1.36	nd	nd	nd	nd
D2. gallic acid	37.29 ± 2.32	nd	nd	nd	nd
D3. gallic acid	14.82 ± 0.36	nd	nd	nd	nd
Galloylquinic acid	201.08 ± 12.26	nd	nd	nd	nd
D4. gallic acid	37.92 ± 1.67	nd	nd	nd	nd
Methyl gallate	2848.28 ± 69.12	111.72 ± 7.09	14.13 ± 1.64	12.11 ± 0.41	12.92 ± 0.97
Methyl digallate ester	2341.91 ± 56.43	75.84 ± 5.28	12.70 ± 0.66	11.92 ± 0.32	12.95 ± 0.99
Trigalloyl glucose	499.75 ± 12.93	nd	nd	nd	nd
Tetragalloyl glucose	604.19 ± 12.44	nd	nd	nd	nd
Pentagalloyl glucose	1001.18 ± 32.40	nd	nd	nd	nd
Hexagalloyl glucose	496.01 ± 16.02	nd	nd	nd	nd
Phenolic acids					
Ellagic acid	97.89 ± 3.45	nd	nd	nd	nd
*p*-hydroxybenzoic acid	9.57 ± 2.36	nd	9.98 ± 1.30	11.74 ± 1.47	5.74 ± 0.24
Coumaric acid glucoside	nd	nd	7.77 ± 0.14	5.03 ± 4.36	8.05 ± 0.13
Coumaric acid glucoside isomer (1)	nd	nd	8.45 ± 0.36	8.32 ± 0.14	8.86 ± 0.18
Coumaric acid glucoside isomer (2)	nd	nd	7.47 ± 0.07	7.57 ± 0.13	7.45 ± 0.08
Coumaric acid glucoside isomer (3)	nd	nd	8.88 ± 0.27	8.15 ± 0.24	8.29 ± 0.17
Coumaric acid glucoside isomer (4)	nd	nd	9.03 ± 0.22	8.27 ± 0.25	9.23 ± 0.19
Ferulic acid hexoside (1)	nd	nd	28.28 ± 4.33	21.93 ± 3.06	41.64 ± 2.75
Ferulic acid hexoside (2)	nd	nd	14.06 ± 0.92	8.75 ± 1.82	10.15 ± 1.58
Flavonols					
Quercetin 3-*O*-glucoside	nd	62.30 ± 4.39	nd	nd	nd
Quercetin 3-*O*-rutinoside	nd	49.08 ± 3.27	nd	nd	nd
Quercetin galactoside	nd	18.67 ± 1.36	nd	nd	nd
Quercetin xylose	nd	13.54 ± 1.06	nd	nd	nd
D. Quercetin	nd	3.31 ± 0.78	2.03 ± 0.16	1.86 ± 0.06	1.96 ± 0.15
Quercetin	nd	21.94 ± 3.85	nd	nd	nd
Kaempferol	nd	2.43 ± 0.10	nd	nd	nd
Mangiferin	2.83 ± 0.11	25.36 ± 1.46	1.26 ± 1.45	0.35 ± 0.04	0.46 ± 0.12
Σ Galloyl derivatives	10,029.50	528.87	26.83	24.03	25.88
Σ Phenolic acids	107.46	-	93.92	79.76	99.41
Σ Flavonols	-	171.28	2.03	1.86	1.96
Σ Total phenolic compounds	10,139.79	723.07	124.04	106.01	127.71

* Triplicate extraction; ** Mango-derived flour: KF = kernel; PF = peel; LF = freeze-dried; FF = foam mat drying; CF = conventional dried. ∑ Galloyl derivates, ∑ phenolics acids, ∑ flavonols, and ∑ total phenolic compounds = sum of all individual phenols from gallic acid, phenolics acids, flavonols and phenolic compounds, respectively. nd = not detected. One-way ANOVA comparisons among drying methods are performed only for mango pulp flour samples. Significant differences (*p* < 0.05) are detected for *p*-hydroxybenzoic acid, coumaric acid glucoside isomer, and ferulic acid hexoside.

**Table 2:** Isoprenoid contents of flours obtained from mango dried pulp and by-products, expressed as mg/100 g on dry basis (mean ± SD; n = 3 *).

Mango Flour **	KF	PF	LF	FF	CF
Tocopherol					
β-tocopherol	nd-	0.32 ± 0.08	nd	nd	nd
α-tocopherol	0.44 ± 0.14	1.81 ± 0.41	1.48 ± 0.34	0.63 ± 0.23	1.76 ± 0.33
Pheophytins					
Pheophytin a isomer 1	nd	0.17 ± 0.04	nd	nd	nd
Pheophytin a isomer 2	nd	0.02 ± 0.00	nd	nd	nd
Pheophytin a isomer 3	nd	0.03 ± 0.01	nd	nd	nd
Pheophytin a isomer 4	nd	0.15 ± 0.04	nd	nd	nd
Pheophytin b	nd	0.05 ± 0.02	nd	nd	nd
Pheophytin b isomer 2	nd	0.04 ± 0.02	nd	nd	nd
Carotenoids compounds					
Phytoene	nd	0.01 ± 0.00	0.02 ± 0.01	0.02 ± 0.01	0.03 ± 0.01
ζ-carotene isomer 1	nd	nd	0.08 ± 0.02	0.17 ± 0.01	0.13 ± 0.02
ζ-carotene isomer 2	nd	nd	0.09 ± 0.03	0.18 ± 0.12	0.18 ± 0.03
ζ-carotene isomer 3	nd	0.04 ± 0.01	0.68 ± 0.18	0.79 ± 0.35	0.82 ± 0.12
ζ-carotene isomer 4	nd	nd	0.05 ± 0.01	0.16 ± 0.14	0.05 ± 0.01
ζ-carotene isomer 5	nd	nd	0.14 ± 0.03	0.21 ± 0.09	0.57 ± 0.06
Lutein	nd	0.07 ± 0.02	nd	nd	nd
Zeaxanthin	nd	0.03 ± 0.01	nd	nd	nd
13Z-β-carotene	nd	0.02 ± 0.01	0.04 ± 0.01	0.10 ± 0.01	0.04 ± 0.01
α-carotene	nd	0.06 ± 0.03	0.04 ± 0.01	0.03 ± 0.00	0.06 ± 0.01
9Z-α-carotene	nd	0.02 ± 0.01	0.05 ± 0.01	0.03 ± 0.01	0.06 ± 0.01
β-carotene	0.03 ± 0.01	0.33 ± 0.09	0.50 ± 0.14	0.43 ± 0.18	0.77 ± 0.10
9Z-β-carotene	0.01 ± 0.00	0.07 ± 0.02	0.09 ± 0.02	0.06 ± 0.03	0.15 ± 0.02
Σ Tocopherols	0.44	2.13	1.48	0.63	1.76
Σ Pheophytins	nd-	0.46	nd	nd	nd
Σ Carotenoid compounds	0.04	0.65	1.78	2.18	2.86
Σ Total isoprenoids	0.48	3.24	3.26	2.81	4.62

* Triplicate extraction; ** Mango-derived flour: KF = kernel; PF = peel; LF = freeze-dried; FF = foam mat drying; CF = conventional dried. Σ tocopherols, Σ Pheophytins, Σ carotenoids compounds and Σ isoprenoids = sum of all individuals tocopherols, pheophytins, carotenoids and isoprenoids, respectively. nd = not detected. One-way ANOVA comparisons among mango pulp flours obtained by different drying methods revealed significant differences (*p* < 0.05) for α-tocopherol, ζ-carotene isomer 5, α-carotene, 13Z-β-carotene and 9Z-β-carotene.

**Table 3:** Bioaccessibility of phenolic compounds (mg/ 100 g) in mango dried pulp and by-products (mean ± SD; n = 4 *).

Cupcakes **	K-CK		P-CK		L-CK		F-CK		C-CK	
	Undigested	Bioaccessible Fraction	Undigested	Bioaccessible Fraction	Undigested	Bioaccessible Fraction	Undigested	Bioaccessible Fraction	Undigested	Bioaccessible Fraction
Galloyl derivates										
Galloyl glucose	494.74 ± 70.59	35.32 ± 0.80	143.50 ± 19.17	30.18 ± 0.68	nd	nd	nd	nd	nd	nd
Gallic acid	601.51 ± 40.12	121.66 ± 14.11	111.06 ± 23.03	7.40 ± 1.05	nd	nd	nd	nd	nd	nd
D1. gallic acid	35.02 ± 8.72	14.43 ± 4.35	nd	nd	nd	nd	nd	nd	nd	nd
D2. gallic acid	31.11 ± 5.16	9.02 ± 2.05	nd	nd	nd	nd	nd	nd	nd	nd
D3. gallic acid	38.44 ± 10.81	7.74 ± 4.73	nd	nd	nd	nd	nd	nd	nd	nd
Galloylquinic acid	47.86 ± 15.54	3.65 ± 0.84	nd	nd	nd	nd	nd	nd	nd	nd
D4. gallic acid	96.04 ± 43.43	3.04 ± 0.14	nd	nd	nd	nd	nd	nd	nd	nd
Methyl gallate	1780.20 ± 629.29	38.37 ± 7.83	53.48 ± 13.59	2.44 ± 0.08	12.78 ± 0.37	2.26 ± 0.05	16.79 ± 3.19	1.41 ± 1.17	13.11 ± 0.88	1.16 ± 1.24
Methyl digallate ester	312.94 ± 139.71	7.87 ± 2.65	16.70 ± 1.58	2.40 ± 0.09	3.44 ± 5.96	0.30 ± 0.84	12.26 ± 1.13	nd	nd	nd
Trigalloyl glucose	120.76 ± 9.77	3.25 ± 0.56	nd	nd	nd	nd	nd	nd	nd	nd
Tetragalloyl glucose	16.84 ± 4.17	3.02 ± 0.15	nd	nd	nd	nd	nd	nd	nd	nd
Pentagalloyl glucose	42.85 ± 20.09	2.71 ± 0.29	nd	nd	nd	nd	nd	nd	nd	nd
Hexagalloyl glucose	17.72 ± 4.25	2.93 ± 0.88	nd	nd	nd	nd	nd	nd	nd	nd
Phenolic acids										
Ellagic acid	63.45 ± 15.11	20.06 ± 1.74	nd	nd	nd	nd	nd	nd	nd	nd
p-hydroxybenzoic acid	7.32 ± 4.14	2.56 ± 0.48	nd	nd	18.77 ± 8.85	51.08 ± 3.44	15.04 ± 5.08	54.12 ± 2.72	26.31 ± 2.34	47.59 ± 1.87
Coumaric acid glucoside	nd	nd	nd	nd	2.14 ± 3.70	1.59 ± 0.13	2.26 ± 3.91	1.88 ± 0.59	9.27 ± 0.31	0.77 ± 0.83
Coumaric acid glucoside isomer (1)	nd	nd	nd	nd	7.17 ± 0.72	1.73 ± 0.12	7.41 ± 0.21	1.72 ± 0.12	8.00 ± 0.17	1.75 ± 0.12
Coumaric acid glucoside isomer (2)	nd	nd	nd	nd	6.72 ± 0.36	1.41 ± 0.02	7.02 ± 0.16	1.67 ± 0.19	6.91 ± 0.05	1.56 ± 0.15
Coumaric acid glucoside isomer (3)	nd	nd	nd	nd	7.21 ± 0.49	1.40 ± 0.04	4.61 ± 4.00	1.23 ± 0.50	6.97 ± 0.13	1.39 ± 0.04
Coumaric acid glucoside isomer (4)	nd	nd	nd	nd	7.21 ± 0.45	2.02 ± 0.04	7.34 ± 0.16	1.96 ± 0.06	7.66 ± 0.09	1.85 ± 0.27
Ferulic acid hexoside (1)	nd	nd	nd	nd	7.73 ± 6.90	0.99 ± 0.09	6.71 ± 0.34	0.88 ± 0.09	14.94 ± 3.16	0.63 ± 0.17
Ferulic acid hexoside (2)	nd	nd	nd	nd	4.65 ± 1.88	1.57 ± 0.14	2.33 ± 0.39	1.37 ± 0.44	3.56 ± 0.39	0.99 ± 0.39
Flavonols										
Quercetin glucoside	nd	nd	19.31 ± 5.24	3.07 ± 0.82	nd	nd	nd	nd	nd	nd
Quercetin 3-rutinoside	nd	nd	13.64 ± 3.19	0.03 ± 0.00	nd	nd	nd	nd	nd	nd
Quercetin galactoside	nd	nd	5.18 ± 1.25	0.03 ± 0.00	nd	nd	nd	nd	nd	nd
Quercetin xylose	nd	nd	3.73 ± 0.80	0.03 ± 0.00	nd	nd	nd	nd	nd	nd
D. Quercetin	nd	nd	0.13 ± 0.00	0.03 ± 0.00	nd	nd	nd	nd	nd	nd
Quercetin	nd	nd	7.82 ± 1.88	0.03 ± 0.00	nd	nd	nd	nd	nd	nd
Kaempferol	nd	nd	0.13 ± 0.00	0.03 ± 0.00	nd	nd	nd	nd	nd	nd
Mangiferin	1.29 ± 0.31	0.21 ± 0.19	4.67 ± 1.10	0.26 ± 0.19	0.24 ± 0.21	0.13 ± 0.04	0.10 ± 0.17	0.04 ± 0.06	0.32 ± 0.02	0.05 ± 0.05
Σ Galloyl derivates	3636.03	253.01	324.74	42.41	16.22	2.56	29.04	1.41	13.11	1.16
Σ Phenolic acids	70.77	22.62	-	-	61.59	61.80	52.71	64.48	83.63	56.52
Σ Flavonols	-	-	49.94	3.22	-	-	-	-	-	-
Σ Total phenolic compounds	3708.08	275.83	379.35	45.89	78.05	64.49	81.86	66.29	97.06	57.73

* Quadruplicate digestion; ** K-CK = cupcake made with mango kernel; P-CK = cupcake made with mango peel; L-CK = cupcake made with lyophilized mango pulp; F-CK = cupcake made with foam mat-dried mango pulp; C-CK = cupcake made with conventional dried mango pulp. ∑ Galloyl derivates, ∑ phenolics acids, ∑ flavonols, and ∑ total phenolic compounds = sum of all individual phenols from gallic acid, phenolics acids, flavonols and phenolic compounds, respectively. nd = not detected. Statistical comparisons between undigested and bioaccessible fractions for each phenolic family are performed using one-way ANOVA. Significant differences are observed at *p* < 0.05.

**Table 4:** Bioaccessibility of tocopherols and carotenoids compounds (mg/ 100 g) of flours obtained from mango dried pulp and by-products (mean ± SD; n = 4 *).

Cupcakes **	P-CK		L-CK		F-CK		C-CK	
	Undigested Samples	Micellar Fraction	Undigested Samples	Micellar Fraction	Undigested Samples	Micellar Fraction	Undigested Samples	Micellar Fraction
Tocopherols								
β-tocopherol	0.32 ± 0.02	0.03 ± 0.00	0.18 ± 0.02	0.02 ± 0.00	0.12 ± 0.02	0.05 ± 0.02	0.17 ± 0.02	0.04 ± 0.01
α-tocopherol	6.87 ± 0.30	1.67 ± 0.15	4.88 ± 0.45	1.23 ± 0.33	4.79 ± 1.33	2.63 ± 0.47	5.83 ± 0.52	2.72 ± 0.55
Carotenoids compounds								
ζ-carotene isomer 1	0.03 ± 0.00	nd	0.08 ± 0.01	nd	0.07 ± 0.02	0.07 ± 0.00	0.10 ± 0.01	0.02 ± 0.00
ζ-carotene isomer 2	nd	nd	nd	nd	nd	-	0.02 ± 0.00	-
ζ-carotene isomer 3	nd	0.03 ± 0.00	nd	nd	nd	0.02 ± 0.00	0.03 ± 0.00	0.02 ± 0.00
Lutein	0.06 ± 0.00	nd	0.03 ± 0.00	nd	0.02 ± 0.00	nd	0.03 ± 0.00	nd
Zeaxanthin	0.06 ± 0.00	nd	0.05 ± 0.01	nd	0.03 ± 0.01	nd	0.04 ± 0.00	nd
13Z-β-carotene	0.05 ± 0.03	0.03 ± 0.00	nd	0.04 ± 0.002	nd	0.06 ± 0.01	nd	0.13 ± 0.01
α-carotene	0.05 ± 0.00	0.01 ± 0.00	nd	0.01 ± 0.00	nd	0.01 ± 0.00	nd	0.02 ± 0.00
β-carotene	0.21 ± 0.01	nd	0.13 ± 0.01	nd	0.12 ± 0.02	nd	0.13 ± 0.01	nd
9Z-β-carotene	0.05 ± 0.01	nd	0.03 ± 0.00	nd	0.02 ± 0.00	nd	0.03 ± 0.00	nd
Σ Tocopherols	7.19	1.70	5.06	1.25	4.87	2.68	6.00	2.76
Σ Carotenoids compounds	0.51	0.07	0.35	0.07	0.29	0.16	0.38	0.19
Σ Tocopherol and carotenoid compounds	7.70	1.77	5.41	1.32	5.16	2.84	6.38	2.95

* Quadruplicate digestion; ** P-CK = cupcake made with mango peel; L-CK = cupcake made with lyophilized mango pulp; F-CK = cupcake made with foam mat-dried mango pulp; C-CK = cupcake made with conventional dried mango pulp; CO-CK = control cupcake. Tocopherols and carotenoids compounds were not detected in K-CK (cupcake made with mango kernel). Σ tocopherols and Σ carotenoids compounds = sum of all individual tocopherols and carotenoids, respectively. nd = not detected. Statistical comparisons between undigested and micellar fractions are performed using one-way ANOVA. Significant differences are observed at *p* < 0.05.

## Data Availability

The original contributions presented in the study are included in the article; further inquiries can be directed to the corresponding author.

## Supplementary Materials

The following supporting information can be downloaded at https://www.mdpi.com/article/10.3390/foods15193527/s1, Table S1: Color parameters (mean ± SD) of dried mango pulp and by-product flours by DigiEye imaging system; Table S2: Bioaccessibility (%) of phenolic compounds from mango dried pulp and by-products; Table S3: Bioaccessibility (%) of tocopherols and carotenoids compounds from mango dried pulp and by-products; Table S4: Color parameters (mean ± SD) of cupcakes made with dried mango pulp and by-product flours by DigiEye imaging system ((e) external and (i) internal colors); Table S5: Results of one-way ANOVA for sensory attributes.

## Abbreviations

The following abbreviations are used in this manuscript:

SDG	Sustainable Development Goal
SSF	Simulated oral fluid
SGF	Simulated gastric fluid
LF	Lyophilized flour
CF	Conventional flour
FF	Foam mat-dried flour
KF	Kernel flour
PF	Peel flour
L-CK	Lyophilized flour cupcake
C-CK	Conventional flour cupcake
F-CK	Foam mat-dried flour cupcake
K-CK	Kernel flour cupcake
P-CK	Peel flour cupcake
CO-CK	Control cupcake
ANOVA	Analysis of variance
GLM	Generalized linear model
