# Supplementary material for: Upcycling of Mango Pulp and By-Products into Functional Flours: Effects on Bioactive Composition and Bioaccessibility in Cupcakes
Source: Foods. 2026 Oct 2;15(19):3527. doi: 10.3390/foods15193527 (PMC13665290; doi:10.3390/foods15193527)
Supplement: Supplementary file 1 [file foods-15-03527-s001.zip › foods-4585707-supplementary.pdf]

Table S1. Color parameters (mean ± SD) of dried mango pulp and by-product flours by DigiEye imaging system.

Mango flour[1]	*KF*	*PF*	*LF*	*FF*	*CF*
L*	67.34 ± 0.25b	68.41 ± 0.50b	78.27 ± 0.24a	77.08 ± 0.55a	51.77 ± 0.99c
a*	7.13 ± 0.03d	7.45 ± 0.22d	11.76 ± 0.24b	10.98 ± 0.15c	21.99 ± 0.19a
b*	18.02 ± 0.04d	31.17 ± 0.40c	43.94 ± 0.29b	43.51 ± 0.49b	47.63 ± 0.57a
C^*_{ab}	19.38 ± 0.03c	32.05 ± 0.43d	45.49 ± 0.32b	44.87 ± 0.52b	52.46 ± 0.59a
h_{ab}	68.42 ± 0.09c	76.55 ± 0.26a	75.01 ± 0.23b	75.84 ± 0.03b	65.22 ± 0.09d

Different letters within the same row indicate statistically significant differences among samples according to Tukey's post hoc multiple-comparison test ($p < 0.05$).

[1]Mango-derived flour: KF = kernel; PF = peel; LF= freeze dried; FF= Foam mat drying; CF = Conventional dried.

Table S2. Bioaccessibility (%) of phenolic compounds from mango dried pulp and by-products.

*Cupcakes**	*K-CK*	*P-CK*	*L-CK*	*F-CK*	*C-CK*
Galloyl glucose	7.14	21.03	0.00	0.00	0.00
Gallic acid	20.23	6.66	0.00	0.00	0.00
D1. gallic acid	41.21	0.00	0.00	0.00	0.00
D2. gallic acid	29.01	0.00	0.00	0.00	0.00
D3. gallic acid	20.13	0.00	0.00	0.00	0.00
Galloylquinic acid	7.62	0.00	0.00	0.00	0.00
D4. gallic acid	3.16	0.00	0.00	0.00	0.00
Methyl gallate	2.16	4.56	17.70	8.41	8.88
Methyl digallate ester	2.52	14.36	8.63	0.00	0.00
Trigalloyl glucose	2.69	0.00	0.00	0.00	0.00
Tetragalloyl glucose	17.91	0.00	0.00	0.00	0.00
Pentagalloyl glucose	6.33	0.00	0.00	0.00	0.00
Hexagalloyl glucose	16.56	0.00	0.00	0.00	0.00
Phenolic acids					
Ellagic acid	31.61	0.00	0.00	0.00	0.00
p-hydroxybenzoic acid	35.04	0.00	272.09	359.77	180.85
Coumaric acid glucoside	0.00	0.00	74.26	83.14	8.32
Coumaric acid glucoside isomer (1)	0.00	0.00	24.10	23.20	21.92
Coumaric acid glucoside isomer (2)	0.00	0.00	21.06	23.78	22.58
Coumaric acid glucoside isomer (3)	0.00	0.00	19.47	26.69	19.87
Coumaric acid glucoside isomer (4)	0.00	0.00	28.06	26.76	24.13
Ferulic acid hexoside (1)	0.00	0.00	12.85	13.16	4.21
Ferulic acid hexoside (2)	0.00	0.00	33.78	59.00	27.69
Flavonols					
Quercetin glucoside	0.00	15.88	0.00	0.00	0.00
Quercetin 3-rutinoside	0.00	0.19	0.00	0.00	0.00
Quercetin galactoside	0.00	0.49	0.00	0.00	0.00
Quercetin xylose	0.00	0.69	0.00	0.00	0.00
D. Quercetin	0.00	20.15	0.00	0.00	0.00
Quercetin	0.00	0.33	0.00	0.00	0.00
Kaempferol	0.00	20.15	0.00	0.00	0.00
Mangiferin	15.95	5.52	53.81	42.65	14.17
Σ Galloyl derivates	6.96	13.06	15.77	4.86	8.88
Σ Phenolic acids	31.97	0.00	100.34	123.00	67.59
Σ Flavonols	0.00	6.45	0.00	0.00	0.00
Σ Total phenolic compounds	7.44	12.10	82.62	80.99	59.48

*K-CK = cupcake made with mango kernel; P-CK = cupcake made with mango peel; L-CK = cupcake made with Lyophilized mango pulp; F-CK = cupcake made with Foam mat drying mango pulp; C-CK = cupcake made with Conventional dried mango pulp.

∑ Galloyl derivates, ∑ phenolics acids, ∑ flavonols, and ∑ Total phenolic compounds = sum of all individual phenols from gallic acid, phenolics acids, flavonols and phenolic compounds, respectively.

Table S3. Bioaccessibility (%) of tocopherols and carotenoids compounds from mango dried pulp and by-products.

*Cupcakes**	*P-CK*	*L-CK*	*F-CK*	*C-CK*
Tocopherols				
β-tocopherol	9.38	11.11	41.67	23.53
α-tocopherol	24.31	25.20	54.91	46.66
Carotenoids compounds				
ζ-carotene isom 1	0.00	5.00	100.00	20.00
ζ-carotene isom 2	0.00	0.00	0.00	0.00
ζ-carotene isom 3	0.00	66.67	100.00	66.67
Lutein	0.00	0.00	0.00	0.00
Zeaxanthin	0.00	0.00	0.00	0.00
13Z-β-carotene	60.00	0.00	0.00	0.00
α-carotene	20.00	0.00	0.00	0.00
β-carotene	0.00	0.00	0.00	0.00
9Z-β-carotene	0.00	0.00	0.00	0.00
Σ Tocopherols	23.64	24.70	55.03	46.00
Σ Carotenoid compounds	13.73	20.00	55.17	50.00
Σ Tocopherols and carotenoid compounds	22.99	24.40	55.04	46.24

* P-CK = cupcake made with mango peel; L-CK = cupcake made with Lyophilized mango pulp; F-CK = cupcake made with Foam mat drying mango pulp; C-CK = cupcake made with Conventional dried mango pulp. Tocopherols and carotenoids compounds were not detected in K-CK = cupcake made with mango kernel.

Σ tocopherols and Σ carotenoids compounds = sum of all individuals tocopherols and carotenoids. respectively.

Table S4. Color parameters (mean ± SD) of cupcakes made with dried mango pulp and by-product flours by DigiEye imaging system ($_{(e)}$ external and $_{(i)}$ internal colors).

*Cupcakes**	*CO-CK*	*K-CK*	*P-CK*	*L-CK*	*F-CK*	*C-CK*
**L*$_{(e)}$**	79.71 ± 7.79a	61.60 ± 1.30b	64.81 ± 1.50c	74.27 ± 6.06d	72.01 ± 5.25d	60.32 ± 3.77e
**a*$_{(e)}$**	13.75 ± 6.51a	9.18 ± 1.28a	11.97 ± 0.85a	12.76 ± 4.73a	15. 15 ± 5.00a	19.42 ± 3.69a
**b*$_{(e)}$**	40.61 ± 3.38a	22.56 ± 1.42c	35.80 ± 0.56b	37.09 ± 4.65a	40.94 ± 1.65a	39.85 ± 1.02a
**C*$_{ab(e)}$**	43.09 ± 5.13a	24,36 ± 1,80c	37, 75 ± 0,61b	39,31± 5,84b	43,80± 2,84a	44,40 ± 2,35a
**h$_{ab(e)}$**	71.84 ± 7,05a	67,93 ± 1,50a	71,50 ± 1,24a	71,46 ± 4,72a	69,92 ± 5,86a	64,14 ± 3,92a
**L*$_{(i)}$**	85.06 ± 1.60a	49.03 ± 3.27f	60.45 ± 0.80e	72.93 ± 0.72b	70.66 ± 3.28c	65.19 ± 0.87d
**a*$_{(i)}$**	7.98 ± 0.48d	7.54 ± 0.66d	12.15 ± 0.35b	10.86 ± 0.93c	13.30 ± 2.01a	13.87 ± 1.11a
**b*$_{(i)}$**	38.34 ± 0.37c	18.63 ± 1.11d	35.62 ± 0.38c	35.74 ± 1.13c	40.70 ± 1.13a	37.48 ± 1.67b
**C*$_{ab(i)}$**	39.16 ± 0.43b	20,10 ± 1,23c	37,63 ± 0,42b	37,35 ±1,33b	42,86 ± 0,44a	39,97 ± 1,91b
**h$_{ab\ (i)}$**	78.25 ± 0.63a	67,98 ± 1,21d	71,17 ± 0,46c	73,11 ± 0,94b	71,88 ± 3,03c	69,71 ± 0,87c

Different letters within the same row indicate statistically significant differences among samples according to Tukey's post hoc multiple-comparison test ($p < 0.05$).*Cupcake Mango-derived flour: K-CK = kernel; P-CK = peel; L-CK= freeze dried; F-CK= Foam mat drying; C-CK = Conventional dried.CO-CK = Control cupcake.

Table S5. Results of one-way ANOVA for sensory attributes.

Attribute	*F-value*	*p-value*
Surface color	5,08	0,000
Interior color	3,03	0,016
Aroma	0,72	0,610
Taste	4,15	0,002
Texture	3,73	0,005

F and *p* values correspond to the one-way ANOVA performed for each sensory attribute. Statistical significance was established at $p < 0.05$.
